# Extended treatment for venous thromboembolism: Is direct oral anticoagulant dose‐reduction better for all?

**DOI:** 10.1002/hem3.70327

**Published:** 2026-03-10

**Authors:** Mandy N. Lauw, Saskia Middeldorp, Marc Carrier

**Affiliations:** ^1^ Department of Hematology Erasmus University Medical Center Rotterdam The Netherlands; ^2^ Department of Internal Medicine Radboudumc Nijmegen The Netherlands; ^3^ Department of Medicine Ottawa Hospital Research Institute University of Ottawa Ottawa Ontario Canada

Treatment for acute venous thromboembolism (VTE) requires a minimum of 3 months of therapeutic anticoagulation to prevent recurrence.^1^ Guidelines recommend extending anticoagulation beyond this initial period in patients with unprovoked VTE or those with persisting risk factors such as active cancer.^2^ Following the introduction of direct oral anticoagulants (DOACs) for acute VTE management, two clinical trials investigated extended treatment with reduced‐dose apixaban (2.5 mg twice daily)^3^ or rivaroxaban (10 mg once daily) in patients with VTE at intermediate risk of recurrence.^4^ While both trials showed that apixaban and rivaroxaban were more effective than placebo or aspirin, respectively, in preventing recurrent VTE, they were not designed or powered to compare reduced‐dose with full‐dose regimens. Although the results suggested that extended treatment with reduced‐dose DOACs may be as effective and potentially safer as full‐dose therapy, adequately designed and powered trials were awaited to address this important knowledge gap.

In the past 2 years, three randomized controlled trials (RCTs) have consolidated the efficacy and safety of reduced‐dose DOACs, including in patients at high risk of recurrent VTE. The RENOVE study investigated patients with acute symptomatic pulmonary embolism or proximal deep vein thrombosis at high risk of recurrent VTE, defined as first unprovoked VTE, recurrent VTE, or with other persistent risk factors.^5^ After completing 6–24 months of full‐dose anticoagulation, patients were randomized to receive reduced‐ or full‐dose apixaban or rivaroxaban. Although RENOVE did not meet formal noninferiority for reduced‐dose versus full‐dose DOACs, the absolute difference in 5‐year cumulative outcomes favored reduced dosing with a clear net clinical benefit (recurrent VTE [+0.4%] vs. clinically relevant bleeding [CRB, the composite outcome of major bleeding and clinically relevant non‐major bleeding] [−5.3%]). The EVE and API‐CAT trials included patients with cancer‐associated VTE, comparing reduced‐ versus full‐dose apixaban for extended anticoagulation after at least 6 months of full‐dose therapy.^6^, ^7^ They showed that reduced‐dose apixaban was noninferior to full‐dose apixaban for preventing recurrent VTE, while significantly lowering major and CRB.^7^

Collectively, these studies suggest a net clinical benefit for extended treatment with reduced‐dose DOACs among patients at high risk of recurrent VTE including those with active cancer (Table 1).^8^

The key question now is whether reduced‐dose DOACs should become the standard for all patients receiving extended anticoagulation for VTE, or whether certain populations warrant a more individualized approach? Moreover, are there any differences between DOACs?

**Table 1:** Summary of characteristics and outcomes of recent trials on extended treatment of venous thromboembolism (VTE).

	**RENOVE (2025)**	**EVE (2024)**	**API‐CAT (2025)**
**Study design**	Multicenter, randomized, open‐label, blinded endpoint, noninferiority design	Multicenter, randomized, double‐blind design	Multicenter, randomized, double‐blind, blinded endpoint, noninferiority design
**Patient population**	Ambulatory adults ≥ 18 years with acute symptomatic first unprovoked VTE, recurrent VTE, and presence of persistent VTE risk factors, or high risk of VTE recurrence; no active cancer	Adults ≥ 18 years with active cancer at study enrollment and acute VTE	Adults ≥ 18 years with active cancer at inclusion and VTE
**VTE types included**	PE or proximal DVT	Proximal or distal lower extremity DVT; upper extremity DVT; PE; cerebral venous sinus thrombosis; splanchnic vein thrombosis (hepatic, portal, splenic, mesenteric, renal, or gonadal)	Proximal DVT of lower limb; symptomatic or incidental PE
**Exclusion criteria**	− Indication for full‐dose anticoagulation other than VTE − Female with HERDOO2 ≤ 1 − Antiphospholipid syndrome − Isolated distal DVT − High bleeding risk − Renal insufficiency − Chronic liver disease or chronic hepatitis − Dual antiplatelet therapy or aspirin >100 mg − Concomitant use of strong CYP3A4 inhibitors or inducers − Active cancer − Pregnancy − Life expectancy < 12 months	− Therapeutic anticoagulation < 6 months or >12 months prior to randomization − Severe liver disease (Child‐Pugh B or C) − Active hepatitis − Mechanical heart valve prosthesis − Bacterial endocarditis − Use of P2Y12 inhibitors − Use of strong CYP3A4 inducers or inhibitors − Atrial fibrillation or other indication for full‐dose anticoagulation other than VTE − Active bleeding or bleeding disorder − Documented anticoagulant failure − Pregnancy or nursing	− VTE at other locations − Mechanical heart valve prosthesis − Bacterial endocarditis − Antiphospholipid syndrome − Atrial fibrillation or other indication for full‐dose anticoagulation other than VTE − Use of P2Y12 inhibitors − Use of strong CYP3A4 inducers or inhibitors − Dual antiplatelet therapy or aspirin > 100 mg − High bleeding risk − ECOG 3 or 4 − Life expectancy < 12 months − Pregnancy or nursing
**Enrollment**	2768 patients	370 patients	1766 patients
**Anticoagulation before randomization**	6–24 months	6–12 months	≥6 months
**Follow‐up duration**	Median 37.1 months (IQR 24.0–48.3)	12 months	12 months
**Treatment arms**	Reduced dose (apixaban 2.5 mg BID or rivaroxaban 10 mg once daily) Full dose (apixaban 5 mg BID or rivaroxaban 20 mg once daily)	Apixaban 2.5 mg BID Apixaban 5 mg BID	Apixaban 2.5 mg BID Apixaban 5 mg BID
**Primary endpoint**	Symptomatic recurrent VTE	Combined rate of major bleeding plus CRNMB	Symptomatic or incidental recurrent VTE
**Key secondary endpoints**	Major bleeding or CRNMB Net clinical benefit (composite of symptomatic VTE, major bleeding, and CRNMB)	Thrombus recurrence (DVT and VTE at other locations), PE, fatal PE, arterial thromboembolism	Major bleeding or CRNMB
**Primary results**	Five‐year cumulative incidence for symptomatic recurrent VTE: Reduced dose: 2.2% (95% CI 1.1–3.3) Full dose: 1.8% (95% CI 0.8–2.7) aHR 1.32 (95% CI 0.67–2.60; P = 0.23 for noninferiority)	One‐year cumulative incidence for composite bleeding outcome: Apixaban 2.5 mg BID: 9.6% Apixaban 5 mg BID: 13.5% HR 0.72 (95% CI 0.38–1.37; P = 0.39)	One‐year cumulative incidence for recurrent VTE: Apixaban 2.5 mg BID: 2.1% Apixaban 5 mg BID: 2.8% aSHR 0.76 (95% CI 0.41–1.41; P = 0.001 for noninferiority)
**Secondary results**	Five‐year cumulative incidence for major bleeding or CRNMB: Reduced dose: 9.9% (95% CI 7.7–12.1) Full dose: 15.2% (95% CI 12.8–17.6) aHR 0.61 (95% CI 0.48–0.79) Five‐year cumulative incidence for net clinical benefit: Reduced dose: 11.8% (95% CI 9.4–14.3) Full dose: 16.5% (95% CI 14.0–19.0) aHR 0.67 (95% CI 0.53–0.86)	One‐year cumulative incidence for recurrent thromboembolism: Apixaban 2.5 mg BID: 5.0% Apixaban 5 mg BID: 4.4% HR 1.00 (95% CI 0.40–2.53; P = 1.0)	One‐year cumulative incidence for major bleeding or CRNMB: Apixaban 2.5 mg BID: 12.1% Apixaban 5 mg BID: 15.6% aSHR 0.75 (95% CI 0.58–0.97; P = 0.03 for superiority)

Abbreviations: aHR, adjusted hazard ratio; aSHR, adjusted subhazard ratio; BID, twice daily; CRNMB, clinically relevant non‐major bleeding; DVT, deep vein thrombosis; ECOG, Eastern Cooperative Oncology Group performance status scale; HR, hazard ratio; IQR, interquartile range; PE, pulmonary embolism; VTE, venous thromboembolism.

## APIXABAN OR RIVAROXABAN

A post hoc analysis of RENOVE showed a cumulative incidence of CRB (median follow‐up of 37 months) of 16.5% with apixaban versus 14.4% with rivaroxaban at full‐dose, and 11.0% versus 9.0%, respectively, at reduced‐dose.^9^ Major bleeding occurred in 4.6% versus 3.5% (full‐dose) and 3.0% versus 1.6% (reduced‐dose). Recurrent VTE rates were low and comparable across groups. Dose reduction consistently lowered CRB risk for both anticoagulants without increasing recurrence. These data suggest that during extended treatment, apixaban and rivaroxaban have similar long‐term safety and efficacy profiles for non‐cancer‐associated VTE, and that reduced dosing provides a favorable risk benefit ratio for both DOACs.

## PATIENTS WITH OBESITY

Another post hoc analysis of RENOVE examined outcomes according to body mass index (BMI). Among patients with obesity (BMI ≥ 30 kg/m2), reduced‐dose DOACs were associated with a higher 5‐year cumulative incidence of recurrent VTE compared with full‐dose therapy (3.6% vs. 0.5%; adjusted hazard ratio [aHR] 6.72, 95% CI 0.99–6.72), and a nonsignificant reduction in CRB (9.4% vs. 13.9%; aHR 0.74, 95% CI 0.47–1.18).^10^ Arterial thrombosis was numerically higher in the reduced‐dose group (aHR 1.73, 95% CI 0.95–3.14). These findings suggest that in patients with obesity, reduction may increase thrombotic risk without improving overall net clinical benefit. These trends were not confirmed in the predefined subgroup analyses of API‐CAT, although few patients had a BMI > 30 kg/m^2^ and even fewer >35 kg/m^2^.^7^ Obesity was also not a predictor of bleeding in API‐CAT.^11^ Consistent with this, a small observational study in patients with severe obesity (BMI > 40 kg/m^2^ or weight >120 kg) found no signal of poorer efficacy or safety outcomes with dose‐reduced apixaban during extended treatment.^12^

## PATIENTS WITH HIGH‐RISK PULMONARY EMBOLISM

In patients with pulmonary embolism at high or intermediate‐high risk, dose reduction of DOACs may also not lead to improvement in net clinical benefit and death. In a post hoc analysis of RENOVE on this subgroup of patients, recurrent VTE occurred in 3.2% of patients receiving reduced‐dose versus 1.9% with full‐dose DOACs (aHR 2.10, 95% CI 0.37–12.0).^10^ CRB was comparable (15.4% vs. 15.5%; aHR 0.77, 95% CI 0.41–1.44), and the composite of recurrence and bleeding showed no significant difference (aHR 0.87, 95% CI 0.49–1.57). All‐cause mortality was also similar between reduced‐ and full‐dose groups (15.0% vs. 13.2%; aHR 0.72, 95% CI 0.36–1.46). In contrast, among patients with intermediate‐low or low‐risk pulmonary embolism, reduced‐dose DOACs were associated with a 48% reduction in bleeding and 43% improvement in net clinical benefit. This supports individualized dosing based on pulmonary severity rather than a uniform reduced‐dose strategy.

## PATIENTS WITH THROMBOPHILIA

RENOVE also evaluated reduced‐dose DOACs in a subanalysis of patients with thrombophilia.^5^ Approximately 37% had non‐major thrombophilia (i.e., isolated heterozygous Factor V Leiden or prothrombin G20210A mutations, elevated Factor VIII), and 17% had “major thrombophilia” (i.e., protein C, S or antithrombin deficiency, homozygous Factor V Leiden or prothrombin G20210A mutation, combined thrombophilia, or presence of antiphospholipid antibodies). In non‐major thrombophilia, recurrent VTE occurred in 4.1% of patients receiving reduced‐dose versus 0% with full‐dose DOACs (aHR 11.7, 95% CI 0.37–375) while, interestingly, there were no recurrent VTE in the 218 patients with major thrombophilia, irrespective of DOAC dose group. CRB was comparable (7.5% vs. 6.8%; aHR 1.14, 95% CI 0.40–3.22) for non‐major thrombophilia but reduced‐dose DOAC showed a beneficial trend for major thrombophilia (6.4% vs. 18.6%; aHR 0.35, 95% CI 0.12–1.00), which also determined the net clinical benefit (non‐major thrombophilia: aHR 1.46, 95% CI 0.56–3.83, major thrombophilia: aHR 0.35, 95% CI 0.12–1.00). Thrombophilia was not considered in EVE or API‐CAT, and patients with antiphospholipid syndrome were excluded from both RENOVE and API‐CAT. Hence, evidence for the efficacy and safety of reduced‐dose DOACs in patients with various types of thrombophilia remains uncertain, as numbers were quite small and observed differences were not statistically significant.

## PATIENTS WITH UNUSUAL SITE VTE OR SPECIFIC TYPES OF CANCER

The exclusion criteria of the trials highlight important patient populations that were underrepresented or not evaluated (Table 1). Both RENOVE and API‐CAT only enrolled patients with pulmonary embolism or (proximal) deep vein thrombosis.^5^, ^7^ In contrast, EVE included a broader spectrum of VTE such as upper extremity deep vein thrombosis, cerebral venous sinus thrombosis, and splanchnic vein thrombosis, although these only accounted for a small proportion of participants (61/360 patients included [16.9%], cerebral or splanchnic vein thrombosis 6.7%).^6^ Hence, the efficacy and safety of reduced‐dose DOACs for extended treatment of VTE at unusual sites remain uncertain in the absence of separate studies.^13^

Similarly, certain cancer subtypes, particularly hematologic or intracranial malignancies, were infrequently included in EVE and API‐CAT. Although extrapolating the observed benefits of reduced‐dose DOACs to these populations may be clinically reasonable, the lack of evidence introduces uncertainty, highlighting the need for dedicated studies in these underrepresented subgroups.

## SUMMARY

Growing evidence has demonstrated the efficacy and improved safety profile of reduced‐dose DOAC for extended treatment of VTE. Post hoc analyses of existing trials provide valuable insights to help clinicians individualize anticoagulation with DOAC dosing during extended VTE treatment. However, these findings should be interpreted with caution as such analyses are inherently hypothesis‐generating, often limited by insufficient statistical power, multiple comparisons, and potential selection bias. The evidence for reduced‐dose DOACs remains limited for certain populations including patients with obesity (≥30 kg/m^2^), thrombophilia, unusual site VTE, and specific types of cancer (Table 2). In these populations, the balance between thrombotic and bleeding risks may differ. Therefore, individualized treatment decisions and shared decision‐making should guide the approach to extended anticoagulation for these patients, and DOAC dose‐reduction should not be uniformly applied to all.

**Table 2:** Population considerations for application of direct oral anticoagulant (DOAC) dose‐reduction.

Patients with acute VTE at high risk of recurrence and anticipated indication for long‐term anticoagulation, treated with full‐dose anticoagulation for at least 6 months	
*Populations with demonstrated evidence for the net clinical benefit of DOAC dose‐reduction*	*Populations with limited evidence for the net clinical benefit of DOAC dose‐reduction*
− Patients with (proximal) DVT or PE, with and without active cancer	− Patients with unusual site VTE (cerebral veins, splanchnic veins, Budd‐Chiari syndrome, and other sites)
− Patients with BMI < 30 kg/m^2^	− Patients with obesity (BMI ≥ 30 kg/m^2^)
− Patients with intermediate‐low‐ or low‐risk pulmonary embolism	− Patients with high‐ or intermediate high‐risk pulmonary embolism
− Patients without thrombophilia	− Patients with non‐major^a^ or major thrombophilia,^b^ or antiphospholipid syndrome^c^
− Patients with solid cancers	− Patients with hematologic and intracranial (primary or secondary) malignancies

Abbreviations: BMI, body mass index; DVT, deep vein thrombosis; PE, pulmonary embolism; VTE, venous thromboembolism.

^a^ Isolated Factor V Leiden heterozygosity or isolated prothrombin G20210A gene variant heterozygosity or elevated Factor VIII.

^b^ Protein C, S, and antithrombin deficiency, combined thrombophilia, homozygous Factor V Leiden, or homozygous prothrombin G20210A gene variant.

^c^ Patients with antiphospholipid syndrome were excluded in the RENOVE and API‐CAT trials.

## AUTHOR CONTRIBUTIONS

**Mandy N. Lauw**: Conceptualization; writing—original draft. **Saskia Middeldorp**: Writing—review and editing. **Marc Carrier**: Writing—review and editing.

## CONFLICT OF INTEREST STATEMENT

Dr. Mandy N. Lauw has received research funding from LeoPharma and honoraria from Viatris, Inari Stryker, AbbVie, Amgen, BMS, and Novo Nordisk for advisory or educational activities. All honoraria are paid to her institution.

Prof. Saskia Middeldorp reports honoraria from AstraZeneca, Alveron, Bayer, Hemab, Norgine, Sanofi, Sirius, VarmX, and Viatris for advisory or data safety monitoring boards or educational activities. All honoraria are paid to her institution.

Prof. Marc Carrier has received research funding from Pfizer and honoraria from Anthos, Regeneron, Bayer, Pfizer, BMS, and Leo Pharma. All honoraria are paid to his institution.

## FUNDING

This research received no funding.

## Data Availability

Data sharing is not applicable to this article as no datasets were generated or analyzed during the current study.

## References

[1] Ortel TL , Neumann I , Ageno W , et al. American Society of Hematology 2020 guidelines for management of venous thromboembolism: treatment of deep vein thrombosis and pulmonary embolism. Blood Adv. 2020;4(19):4693‐4738. 10.1182/bloodadvances.2020001830 33007077 PMC7556153

[2] Lyman GH , Carrier M , Ay C , et al. American Society of Hematology 2021 guidelines for management of venous thromboembolism: prevention and treatment in patients with cancer. *Blood Adv*. 2021;5(4):927‐974. Blood Adv. 2021;5(7):1953. 10.1182/bloodadvances.2021004734 33570602 PMC7903232

[3] Agnelli G , Buller HR , Cohen A , et al. Apixaban for extended treatment of venous thromboembolism. N Engl J Med. 2013;368(8):699‐708. 10.1056/NEJMoa1207541 23216615

[4] Weitz JI , Lensing AWA , Prins MH , et al. Rivaroxaban or aspirin for extended treatment of venous thromboembolism. N Engl J Med. 2017;376(13):1211‐1222. 10.1056/NEJMoa1700518 28316279

[5] Couturaud F , Schmidt J , Sanchez O , et al. Extended treatment of venous thromboembolism with reduced‐dose versus full‐dose direct oral anticoagulants in patients at high risk of recurrence: a non‐inferiority, multicentre, randomised, open‐label, blinded endpoint trial. Lancet. 2025;405(10480):725‐735. 10.1016/S0140-6736(24)02842-3 40023651

[6] McBane RD , Loprinzi, C.L. , Zemla T. , et al. Extending venous thromboembolism secondary prevention with apixaban in cancer patients. The EVE trial. J Thromb Haemost. 2024;22(6):1704‐1714. 10.1016/j.jtha.2024.03.011 38537780

[7] Mahé I , Carrier M , Mayeur D , et al. Extended reduced‐dose apixaban for cancer‐associated venous thromboembolism. N Engl J Med. 2025;392(14):1363‐1373. 10.1056/NEJMoa2416112 40162636

[8] Barbosa LM , Oliveira VMR , Araujo B , Nunes MdCP , Moreira HG . Reduced dose versus full dose of direct oral anticoagulation in extended treatment of venous thromboembolism: an updated meta‐analysis of randomized controlled trials. J Thromb Haemost. 2025;23(11):3733‐3737. 10.1016/j.jtha.2025.07.029 40784555

[9] Girard P , Presles E , Sanchez O , et al. Apixaban or rivaroxaban for extended anticoagulation in patients with venous thromboembolism: a post hoc analysis of the RENOVE clinical trial. Blood. 2025;146(suppl 1):740. 10.1182/blood-2025-740

[10] 2025 oral communications abstracts. Res Pract Thromb Haemost. 2025;9:102925. 10.1016/j.rpth.2025.102925

[11] Mahé I , Chapelle C , Girard P , et al. Predictors of clinically relevant bleeding during extended anticoagulation for cancer‐associated venous thromboembolism (API‐CAT): a post‐hoc analysis of a randomised, non‐inferiority trial. Lancet Haematol. 2026;13(1):e41‐e48. 10.1016/S2352-3026(25)00291-1 41365312

[12] Rapoport EA , Townsend NW , Farhat N , Patel P , Billett HH . Dose‐reduced apixaban for secondary prophylaxis in patients with severe obesity: a single center analysis. Blood Adv. 2025;9(19):4776‐4779. 10.1182/bloodadvances.2025016037 40590870 PMC12494910

[13] Delluc A , Carrier M , Lauw M , et al. Indefinite anticoagulation with reduced‐intensity direct oral anticoagulants in patients with splanchnic vein thrombosis. An international practice survey. Blood Coagul Fibrinolysis. 2025;36(8):364‐370. 10.1097/MBC.0000000000001388 41065579

